# CircSETD3 (Hsa_circ_0000567) acts as a sponge for microRNA-421 inhibiting hepatocellular carcinoma growth

**DOI:** 10.1186/s13046-019-1041-2

**Published:** 2019-02-22

**Authors:** Liangliang Xu, Xinfu Feng, Xiangyong Hao, Peng Wang, Yanfang Zhang, Xiaobo Zheng, Lian Li, Shengsheng Ren, Ming Zhang, Mingqing Xu

**Affiliations:** 1 Department of Liver Surgery, West China Hospital, Sichuan University, Sichuan Province, Chengdu, 610041 China; 2 Department of Hepatobiliary Surgery, Guizhou Provincial People’s Hospital, Guiyang, 550000 Guizhou Province China; 3 Department of General Surgery, Gansu Provincial Hospital, Lanzhou, 730000 Gansu Province China; 4 Center of Infectious Diseases, West China Hospital, Sichuan University, Sichuan Province, Chengdu, 610041 China; 5 Department of General surgery, Mianzhu hospital of West China hospital, Sichuan University, Mianzhu City, Sichuan Province China

**Keywords:** Circular RNA, SETD3, microRNA, Hepatocellular carcinoma, Growth

## Abstract

**Background:**

Circular RNAs (circRNAs) play important roles in tumourigenesis and tumour progression. However, the expression profiles and functions of circRNAs in hepatocellular carcinoma (HCC) are largely unclear.

**Methods:**

The expression profiles of circRNAs in HCC were identified through microarray analysis and were validated through quantitative reverse transcription polymerase chain reaction (qRT-PCR). Survival curves were plotted using the Kaplan-Meier method and compared using the log-rank test. The circular structure of candidate circRNA was confirmed through Sanger sequencing, divergent primer PCR, and RNase R treatments. Proliferation of HCC cells was evaluated in vitro and in vivo. The microRNA (miRNA) sponge mechanism of circRNAs was demonstrated using dual-luciferase reporter and RNA immunoprecipitation assays.

**Results:**

CircSETD3 (hsa_circRNA_0000567/hsa_circRNA_101436) was significantly downregulated in HCC tissues and cell lines. Low expression of circSETD3 in HCC tissues significantly predicted an unfavourable prognosis and was correlated with larger tumour size and poor differentiation of HCC in patients. In vitro experiments showed that circSETD3 inhibited the proliferation of HCC cells and induced G1/S arrest in HCC cells. In vivo studies revealed that circSETD3 was stably overexpressed in a xenograft mouse model and inhibited the growth of HCC. Furthermore, we demonstrated that circSETD3 acts as a sponge for miR-421 and verified that mitogen-activated protein kinase (MAPK)14 is a novel target of miR-421.

**Conclusion:**

CircSETD3 is a novel tumour suppressor of HCC and is a valuable prognostic biomarker. Moreover, circSETD3 inhibits the growth of HCC partly through the circSETD3/miR-421/MAPK14 pathway.

**Electronic supplementary material:**

The online version of this article (10.1186/s13046-019-1041-2) contains supplementary material, which is available to authorized users.

## Background

Heaptocellular carcinoma (HCC) is the most common primary hepatic malignancy and is a serious health problem worldwide [1, 2]. It is the fifth most common cancer and the third leading cause of cancer-related deaths globally [3]. Annually, approximately 0.75 million new cases are identified and almost 500,000 patients die because of HCC [4]. Only 30–40% of new HCC cases respond to curative treatments, such as radio frequency ablation, resection and transplantation. Moreover, following curative treatments, the 5-year rate of recurrence or metastases is 70% [5, 6]. Compared with other human cancers, many risk factors for hepatocarcinogenesis have been identified, such as infection with hepatitis B virus and hepatitis C virus, alcohol abuse, cirrhosis caused by inflammation or chronic liver damage, intake of aflatoxin B1 and metabolic syndrome [7–11]. The molecular mechanisms involved in the initiation and progression of HCC are complex and multistep. Further knowledge of these mechanisms would be valuable in predicting prognosis and for the design of more effective therapeutic approaches.

Circular RNA (circRNA) is a novel type of RNA that features a covalently closed loop with no 5′-3′ polarity. CircRNAs tend to be highly expressed in the cytoplasm of eukaryotic cells [12, 13]. CircRNAs are stable, abundant, conserved and are usually expressed in particular tissues or at a specific developmental stage [14, 15]. Since the report that circRNA can act as a “super sponge” for microRNA (miRNA) [13, 14], other studies have revealed that circRNAs broadly participate in the initiation and progression of various diseases, especially in malignant tumours, such as oesophageal squamous cell carcinoma (ciRS-7, circ-ITCH) [16, 17], gastric cancer (circPVT1, circLARP4) [18, 19], colorectal cancer (circITGA7, circCCDC66) [20, 21], and HCC (circMTO1, cSMARCA5) [22, 23]. Recent evidence demonstrated that circRNAs can encode functional peptides or proteins in a splicing-dependent and cap-independent manner, which further clarified the biological function of circRNAs [24, 25]. Previously, we reported that the highly expressed ciRS-7 in HCC tissues is an independent risk factor of microvascular invasion of HCC [26]. However, the full role of circRNAs in HCC remains unclear, so the effects of circRNAs on hepatocarcinogenesis and progression of HCC remain to be investigated.

In the present study, microarray analysis and quantitative reverse transcription polymerase chain reaction (qRT-PCR) were used to demonstrate the significant downregulation of circSETD3 (hsa_circRNA_0000567/hsa_circRNA_101436) in HCC tissues and the close association of circSETD3 with the prognosis of HCC patients who received curative hepatectomy. Furthermore, we found circSETD3 could inhibit the proliferation of HCC cells by targeting the miR-421/mitogen-activated protein kinase (MAPK)14 signalling axis.

## Methods

### Patients and HCC samples

A total of 134 HCC samples were obtained from the clinical sample bank of West China Hospital, Sichuan University. Among them, 58 had paired non-tumorous tissues. The collection of human specimens was approved by the Biomedical Ethics Committee of the West China Hospital, and written informed consent was obtained from each patient. Inclusion criteria for patient selection were curative hepatectomy performed between 2013 and 2014, pathologically diagnosed as HCC by two senior pathologists and no adjunctive treatment prior to the surgery. The exclusion criteria were treatment combined with cholangiocarcinoma or other malignancy and incomplete clinical or prognostic data. All specimens were collected within 15 min after removal from the body and were immediately snap-frozen in liquid nitrogen before storage at − 80 °C. Two pairs of specimens were used for microarray analysis and 56 pairs were used to compare the expression levels of the genes of interest between HCC and paired non-tumorous tissues. All HCC samples, except for the two used for microarray analysis, were used to evaluate the clinical significance and prognostic value of circSETD3.

### CircRNAs microarray hybridization and data analysis

Total RNAs were digested with RNase R (20 U/μl, Epicentre, USA) to remove linear RNAs and enrich circular RNAs. The enriched circular RNAs were amplified and transcribed into fluorescent cRNA utilizing a random priming method (Super RNA Labelling Kit; Arraystar, Rockville, MD, USA). The labelled cRNAs were hybridized onto the Human circRNA Array (8 × 15 K, Arraystar). The slides were incubated for 17 h at 65 °C in a hybridization oven (Agilent, Santa Clara, CA, USA). CircRNAs differentially expressed with statistical significance between HCC and paired normal tissues (fold-change (FC) ≥ 2 and *p* ≤ 0.05) were identified through Volcano Plot filtering. Hierarchical clustering was performed to show the distinguishable expression pattern of circRNAs among samples.

### Genomic DNA and total RNA preparation

Genomic DNA (gDNA) was extracted from tissues and cultured cells using a PureLink™ Genomic DNA Mini Kit (Thermo Fisher Scientific, USA) according to the manufacturer’s protocol. Total RNA was extracted from each specimen using Trizol reagent (Invitrogen, Life Technologies Inc., Germany) according to the manufacturer’s instructions. Row RNA concentration was measured using a ScanDrop Nuclear Acid Analyzer (Analytik Jena AC, Jena, Germany). RNA integration was verified by denatured agarose electrophoresis. If there was no obvious drag band and the peak area of 28S ribosome RNA (rRNA) was approximately twice than that of 18S rRNA, the integrity of total RNA was accepted and used for subsequent experiments [27].

### Quantitative reverse transcription polymerase chain reaction (qRT-PCR)

All primers used for qRT-PCR were designed in primer 5.0 software (Premier, Canada) and synthesized by TsingKe Biotech (Chengdu, China) (Table 1). The complementary DNAs (cDNAs) for circRNA, mRNA, or miRNA measurement were synthesized using random, oligo(dT)_18_ or stem-loop primers, respectively, using RevertAid First Strand cDNA Synthesis Kit (Thermo Fisher Scientific, Waltham, MA, USA). qRT-PCR was performed in triplicate using a Maxima SYBR Green qPCR Master Mix (Thermo Fisher Scientific) on the CFX connect real-time system (Bio-Rad, Hercules, CA, USA). Glyceraldehyde-3-phosphate dehydrogenase (GAPDH) was employed as the intrinsic control for circRNA and mRNA, and U6 was used as the endogenous control for miRNA. Relative expression level of each gene was calculated using the 2^−ΔΔCt^ method.

**Table 1:** All primers used in this study

Gene	Primer	Sequence (5′–3′)
Hsa_circ_0005394	Forward primer	GCTTGATTCTGCACCTGA
	Reverse primer	TACCGCATATTGCTCCTG
Hsa_circ_0001741	Forward primer	GCTAATCGGCGCACAGAA
	Reverse primer	CAGCAAAATAGCATGACTCCAC
Hsa_circ_0006916	Forward primer	TCAACGGGACAGATGATGAAAG
	Reverse primer	CGAGTGCTGAAGATAGGTTGTT
Hsa_circ_0004058	Forward primer	CTTTCCCAGGAACCATTG
	Reverse primer	AAGGTCGGGCTTTAACAT
circSETD3	Forward primer	TGAAGAAGATGAAGTTCGGTAT
	Reverse primer	GTGCCAGATTTCTGAGTTTT
SETD3	Forward primer	GACAGACTCTACGCCATG
	Reverse primer	GTCTCCCAGCAAGTGTTC
GAPDH	Forward primer	ACTCCTCCACCTTTGACGC
	Reverse primer	GCTGTAGCCAAATTCGTTGTC
Divergent GAPDH	Forward primer	GGCCTCCAAGGAGTAAGA
	Reverse primer	GCCCAATACGACCAAATCA
miR-421	Stem-loop Primer	GTCGTATCCAGTGCAGGGTCCGAGGT
		ATTCGCACTGGATACGACGCGCCC
	Forward primer	CTCACTCACATCAACAGACATTAATT
	Reverse primer	GTGCAGGGTCCGAGGT
MAPK14	Forward primer	GAACAAGACAATCTGGGAGGTG
	Reverse primer	TTCGCATGAATGATGGACTGAA
U6	Forward primer	CTCGCTTCGGCAGCACA
	Reverse primer	AACGCTTCACGAATTTGCG

### Circular structure confirmation

The circular structure of circSETD3 was confirmed by Sanger sequencing, divergent primer PCR and RNase R treatment. PCR products amplified by divergent primers of circSETD3 were inserted into the T vector and delivered to Tsingke Biotech for Sanger sequencing. The results were crosschecked with the back-spliced region of circSETD3 supplied by circBASE [28]. In addition, since circRNAs arises normally from pre-mRNA, divergent and convergent primers were used to amplify the circular and linear transcripts of SETD3 in both cDNA and gDNA from HCC tissue, paired non-tumorous tissue and Hep3B cells, respectively. Theoretically, the circular transcript of SETD3 could be only amplified by divergent primers in cDNA not in gDNA. GAPDH was employed as the negative control. For RNase R treatment, 3 μg total RNA extracted from HCC, paired non-tumourous tissue and HepG2 cells were respectively incubated with 10 U RNAse R (20 U/μl, Epicentre, Madison, WI, USA) in a 10 μl volume at 37 °C for 45 min, followed by 70 °C for 10 min to deactivate the RNase R. The treated RNAs were used for RT- PCR.

### Cell culture

All cell lines used in this study (HepG2, Hep3B, Huh7, HCCLM3, SK-Hep1, PLC, LO2, and 293 T) were purchased from the Cell Bank of Type Culture Collection (Chinese Academy of Sciences, Shanghai, China). All cells were cultured in DMEM/high glucose medium (Hyclone, Logan, UT, USA) supplemented with 10% foetal bovine serum (PAN-Biotek, Aidenbach, Bavaria) and 1% penicillin-streptomycin (Hyclone) in a humidified atmosphere at 37 °C containing 5% CO_2_. The authenticity of the cell lines was verified by DNA fingerprinting before use.

### Small interfering (si)RNA and circSETD3-overexpressing lentivirus

siRNA targeting the junction region of the circSETD3 sequence and miR-421 mimics or inhibitors were designed and synthesized by RiboBio (Guangzhou, China). The sequence of circSETD3 siRNA was 5′-CAUCCAGUCAGAAAAAUGGdTdT-3′. The circSETD3-overexpressing plasmid synthesized by Geneseed (Guangzhou, China) was used to co-transfect 293 T cells using two lentivirus packaging plasmids (psPAX2 and pMD2.G). The virus-containing supernatant was collected after 48 h of incubation.

### CCK-8 assay

Cell proliferation was assessed using the Cell Counting Kit (CCK)-8 assay (Beyotime Biotechnology, Nantong, China). Cells (2 × 10^3^) were seeded into each well of 96-well plates. 10 μl of CCK-8 solution was added to each well at six time points. After 1.5 h of incubation at 37 °C, the absorbance at 450 nM was measured using Spectra Max 250 spectrophotometer (Molecular Devices, Sunnyvale, CA, USA). Experiments were independently performed in triplicate.

### Colony formation assays

For the colony formation assays, cells (1 × 10^3^) were suspended and plated into each well of 6-well plates. After 14 days incubation at 37 °C in a chamber with an atmosphere of 5% CO_2_, colonies were fixed with 500 μl of 4% paraformaldehyde (Solarbio, Beijing, China) for 30 min and were stained with crystal violet (Beyotime Biotechnology, Nantong, China) for 25 min. Colonies were counted after photographing the sample (Nikon, Tokyo, Japan).

### 5-Ethynyl-2′-deoxyuridine (EdU) incorporation assays

EdU assays were performed with a Cell-Light EdU DNA Cell Proliferation Kit (RiboBio, Guangzhou, China). The treated cells (3 × 10^4^) were seeded into each well of 24-well plates and incubated for 48 h. After incubation with 300 ul EdU for 2 h, the cells were fixed with 500 μl of 4% paraformaldehyde (Solarbio) and stained with Apollo Dye Solution. Nucleic acid was stained using Hoechst stain. Images were obtained with an inverted fluorescence microscope (Carl Zeiss, Jena, Germany). The proportion of EdU-positive cells was determined.

### Cell cycle and apoptosis assay

To assess the cell cycle and apoptosis, 3 × 10^5^ treated cells were seeded into 6-well plates and cultured for 48 h at 37 °C. The cells for cell cycle analysis were digested using trypsin (Hyclone), washed twice with phosphate-buffered saline (PBS), and fixed in 70% ethanol overnight at 4 °C. The cells were centrifuged at 500 g for 5 min, washed twice with cold PBS, and centrifuged. After treating with RNase A (0.1 mg/ml) and propidium iodide (PI, 0.05 mg/ml) purchased from 4A Biotech (Beijing, China) for 30 min at 37 °C, cell cycle analysis was performed through fluorescence-activated cell sorting flow cytometry (Beckman Coulter, Palo Alto, CA, USA).

For the analysis of apoptosis, cells were trypsinised followed by two PBS washing steps. The cells were stained using the Annexin V/PI detection kit (4A Biotech, Beijing, China) for 5 min at room temperature. The apoptotic cells were measured using flow cytometry (Beckman Coulter). All experiments were repeated at least three times.

### Dual-luciferase assay

293 T cells were seeded in 96-well plates at a density of 1 × 10^4^ cells per well for 24 h before co-transfection. The cells were co-transfected with dual-luciferase reporter vector and miRNA mimics or inhibitors using the Lipofectomine 3000 transfection reagent (Invitrogen, Carlsbad, CA, USA). After 48 h of incubation, firefly and Renilla luciferase activities were measured using a dual-luciferase reporter assay system (Promega) according to the manufacturer’s instructions.

### RNA immunoprecipitation (RIP) assay

The RIP assay was carried out using a Magna RIP RNA Binding Protein Immunoprecipitation Kit (Bersinbio, Guangzhou, China) according to the manufacturer’s protocol. Huh7 cells (2 × 10^7^) were lysed in complete RIP lysis buffer and the cell lysates were divided into two equal parts and incubated with either 5 μg human anti-Argonaute2 (AGO2) antibody (Millipore, Billerica, MA, USA) or non-specific anti-IgG antibody (Millipore) with rotation at 4 °C overnight. Magnetic beads were added to the cell lysates and incubation was continued at 4 °C for 1 h. The samples were then incubated with Proteinase K at 55 °C for 1 h. The enriched RNA was obtained using RNA Extraction Reagent (Enol: Chloroform: Isoamylol = 125:24:1, pH<5.0; Solarbio). The purified RNA was used to detect the expression levels of the genes of interest by qRT-PCR.

### Xenograft nude mouse model

Six-week-old male BALB/C nude mice purchased from Huafukang Bioscience (Beijing, China) were maintained under specific pathogen-free conditions with a 12-h light/dark cycle. All animal experiments were performed in accordance with the guidelines for the Care and Use of Laboratory Animals of Sichuan University. Huh7 cells stably transfected with circSETD3 overexpressing lentivirus or control lentivirus were subcutaneously injected into the right upper back of the nude mice (1 × 10^6^ cells per mouse). Six weeks later, the mice were sacrificed and tumour tissues were collected for examination of the parameters of interest.

### Western blot analysis

The following antibodies were used for western blotting experiments: SETD3 (1:1000 dilution, Novus, St. Charles, MO, USA), MAPK14 (1:1000 dilution, Abcam, Cambridge, UK), CyclinD1 (1:1000 dilution, Cell Signaling Technology, Beverly, MA, USA), PCNA (1:1000 dilution, BosterBio, Wuhan, China), and GAPDH (1:2000 dilution, Zen BioScience, Chengdu, China). Cells were collected from different treatment groups and lysed in RIPA buffer (Beyotime Biotechnology, Nantong, China) containing 1% protease inhibitor (Cell Signaling Technology). The protein concentration was measured using the BCA Protein Assay kit (Beyotime Biotechnology, Nantong, China). Total protein (50 μg) was separated using 10% sodium dodecyl sulphate polyacrylamide gel electrophoresis (SDS-PAGE) and the resolved proteins were transferred to a polyvinylidene difluoride (PVDF) membrane (Biosharp, Anhui, China). After blocking with 5% nonfat dry milk in Tris buffered saline-Tween (TBST) buffer at room temperature for 1 h, the membrane was incubated with primary antibody at 4 °C overnight. The membrane was washed three times (10 min each) using TBST buffer and further incubated with secondary antibody (1:2000 dilution, Zenbio, Chengdu, China) at room temperature for 1 h. The protein bands were scanned in the Chemical Mp Imaging System (Bio-Rad) after treatment with SuperSignal West Femto Agent (Millipore). The band intensity was quantified using the Image J software (NIH, Bethesda, MD, USA) and the expression levels of target proteins were expressed as a ratio of the expression of GAPDH.

### Immunohistochemistry (IHC) assay

Fresh samples were cut to an appropriate size and fixed in 4% paraformaldehyde for 24 h. The fixed specimens were dehydrated in a graded series of ethanol solutions, embedded in paraffin and cut at a thickness of 4 μm. The sections were dewaxed and rehydrated using xylene and ethanol, and high-pressure heat was applied for antigen retrieval. The sections were incubated with the first antibody (MAPK14, 1:100 dilution, Abcam; Ki-67, 1:100 dilution, Millipore; PCNA, 1:50 dilution, Boster; Cyclin D1, 1:150 dilution, Cell Signaling Technology) overnight at 4 °C. Finally, all sections were dehydrated, cleared, mounted, and visualised with a diaminobenzidine-based colorimetric method.

### Statistical analyses

All statistical analyses were performed using SPSS version 21.0 (IBM SPSS Inc., Chicago, IL, USA) and Prism version 5.0 (GraphPad Software, LaJolla, CA, USA) softwares. Categorical variables were expressed as a count or percentage and tested using χ^2^ or Fisher’s exact test, as appropriate. Continuous data are reported as mean ± standard deviation (SD) and compared using Student’s *t* test, the one-way analysis of variance (ANOVA) test or Mann-Whitney test as appropriate. Correlations were calculated using Pearson’s correlation analysis. The cut-off value used to stratify patients into high and low expression groups was the median expression of target genes. Survival curves were plotted using the Kaplan-Meier method and compared using the log-rank test. All tests were 2-sided, and *P* < 0.05 was considered statistically significant.

## Results

### Profiles of circRNAs in HCC

**Fig. 1:**
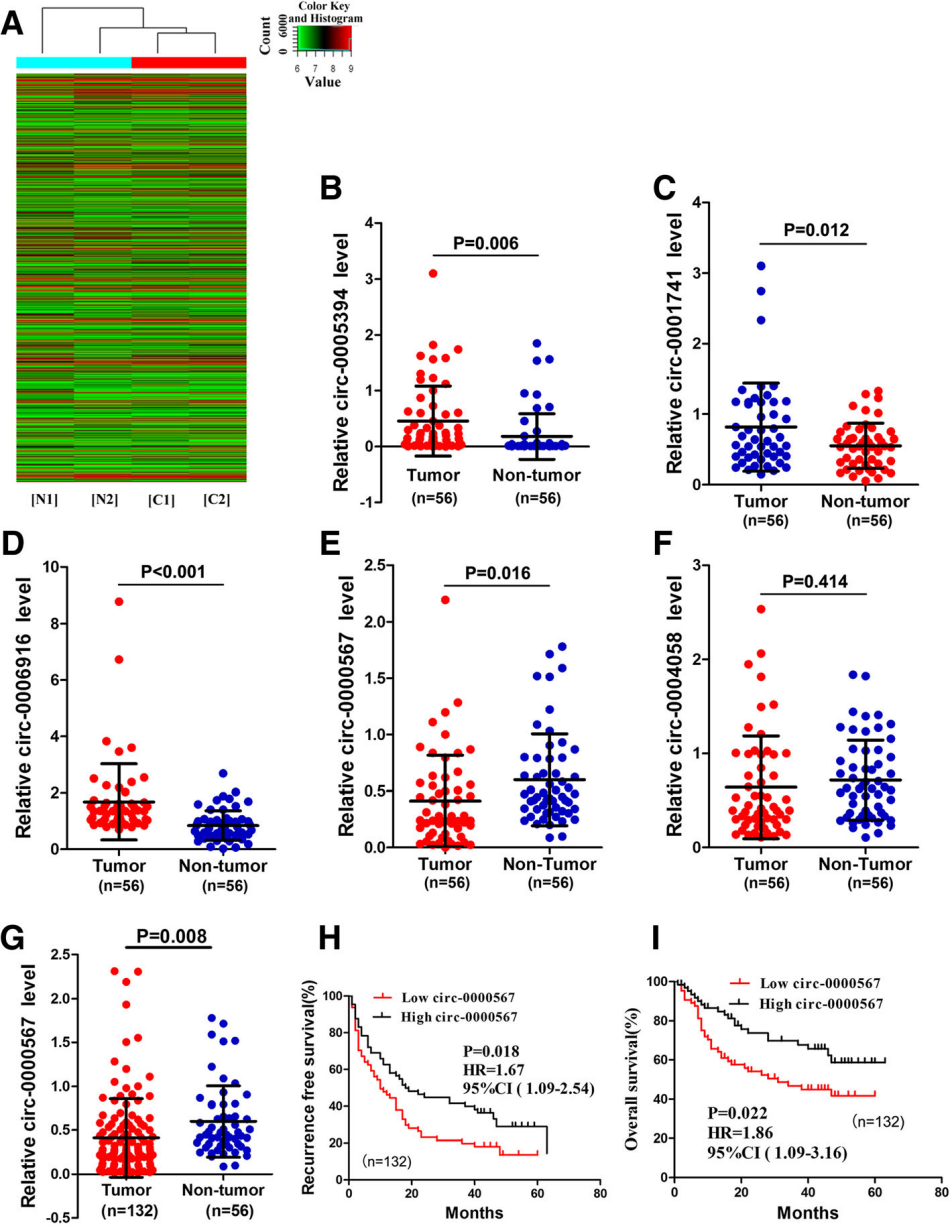
Identification of hsa_circ_0000567 as a candidate biomarker of HCC. **a** Clustered heat map showed differentially expressed circRNAs in two paired HCC (T) and adjacent non-tumorous tissues (N). **b-f** The relative expression of three most upregulated circRNAs (hsa_circ_0005394, hsa_circ_0001741, hsa_circ_0006916) and two most downregulated circRNAs (hsa_circ_0000567, hsa_circ_0004058) were validated by qRT-PCR. **g** The relative expression of hsa_circ_0000567 in 132 HCC and 56 non-tumorous tissues. **h** and **i** Kaplan-Meier’s survival curves depicted the correlations between hsa_circ_0000567 and RFS or OS of HCC patients (the patients were stratified into two groups according to the median of hsa_circ_0000567). Log-rank test was used. For **b-f**, data are presented as means ± SD. HCC, hepatocellular carcinoma; T, tumor tissue; N, non-tumorous tissue; qRT-PCR, quantitative reverse transcription polymerase chain reaction; RFS, recurrence-free survival; OS, overall survival

A total of 4266 circRNAs were detected in HCC and paired non-tumorous samples by the circRNA microarray analysis (Fig. 1a). Among them, 70 circRNAs were significantly aberrantly expressed (*P* < 0.05 and fold-change > 2.0) between HCC tissues and paired non-tumorous tissues. Of the 70 circRNAs, 58 were significantly upregulated and 12 were significantly downregulated in HCC tissues compared with paired adjacent normal tissues. The three most upregulated circRNAs (hsa_circ_0005394, hsa_circ_0001741, and hsa_circ_0006916) and two most downregulated circRNAs (hsa_circ_0000567 and hsa_circ_0004058) were selected and validated by qRT-PCR using 56 HCC and paired non-tumorous tissue samples. As shown in Fig. 1b-f, except for hsa_circ_0004058, the circRNAs displayed a consistent expression level between the microarray and qRT-PCR analyses.

To screen candidate circRNAs for further study, we next analysed the relationship between the expression level of these circRNAs in HCC tissue and the postoperative recurrence-free survival (RFS) of HCC patients. The aforementioned 56 HCC patients were stratified into low and high groups based on the median value of five measured circRNAs. The survival analyses showed that only the low expression of hsa_circ_0000567 was significantly associated with poor RFS (Additional file 1: Figure S1). Next, the relative expression of hsa_circ_0000567 was measured in the 76 other HCC samples. The unpaired *t* test showed that the expression level of hsa_circ_0000567 in all 132 HCC tissues were still lower than that in 56 non-tumorous tissues (Fig. 1g). These patients were similarly stratified into high and low groups based on the median value of hsa_circ_0000567 expression. Survival analyses of these patients revealed that RFS and overall survival (OS) rates of HCC patients in the low hsa_circ_0000567 expression group were significantly lower than patients in the high hsa_circ_0000567 expression group (Fig. 1h and i).

### Confirmation of circular structure of hsa_circ_0000567 (circSETD3)

**Fig. 2:**
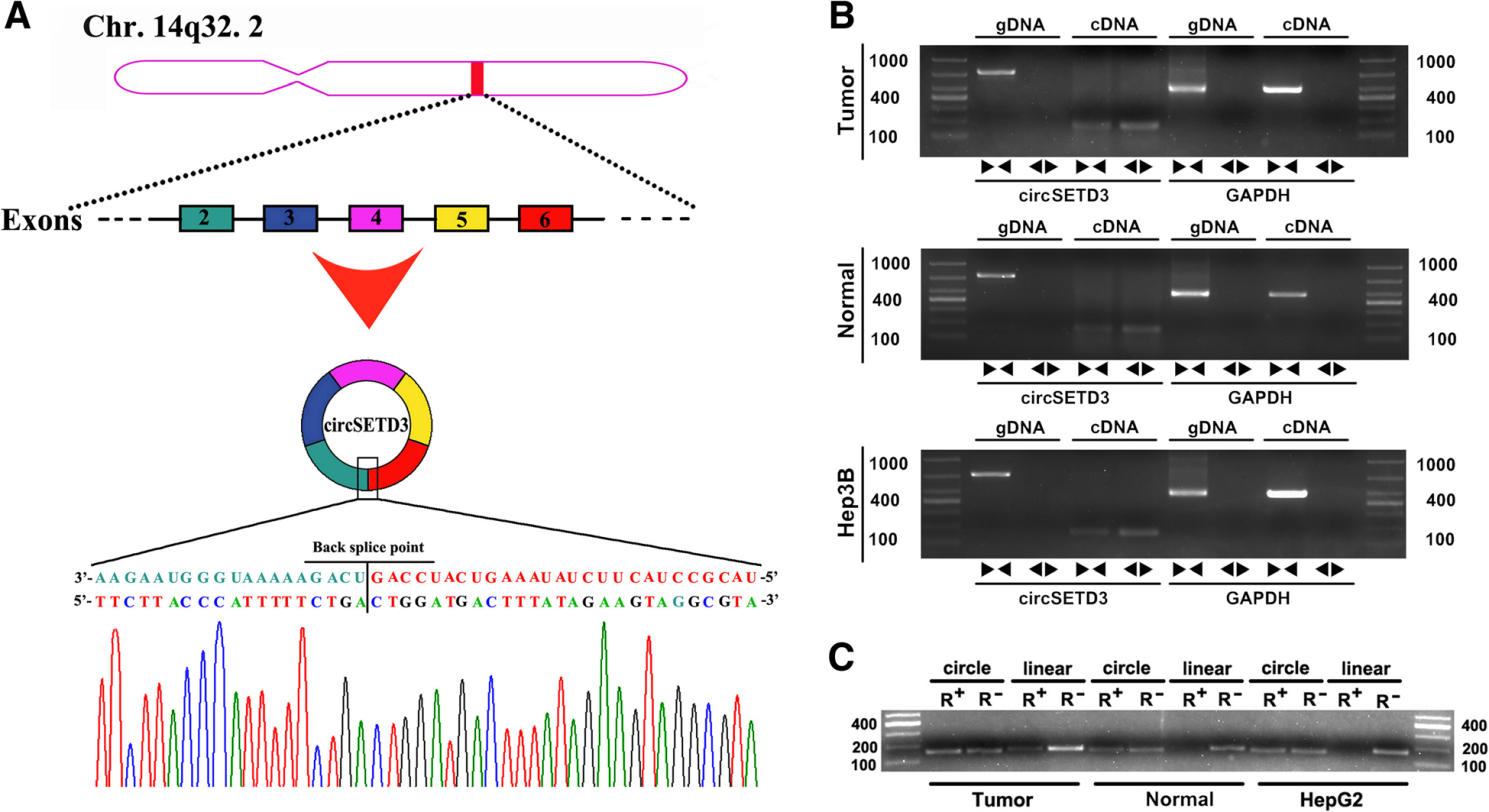
Confirmation of the circular structure of circSETD3. **a** Schematic illustration showed that circSETD3 is located at chromosome 14q32.2 and cyclized from exons 2–6 of SETD3, the PCR products of circSETD3 were confirmed by Sanger sequencing. **b** The existence of cricSETD3 was validated in HCC and paired non-tumorous tissues as well as Hep3B cells. Divergent primers detected circular RNAs in cDNA but not in gDNA. GAPDH was used as negative control. **c** PCR for detecting circSETD3 and SETD3 linear form RNA in HCC and paired non-tumorous tissues as well as HepG2 cells treated with or without RNase R digestion, circSETD3 was resistant to RNase R treatment. SETD3, SET domain-containing 3; cDNA, complementary DNA; gDNA, genomic DNA. PCR, polymerase chain reaction. ►◄, convergent primer; ◄►, divergent primer

Hsa_circ_0000567 was derived from exons 2–6 of SET domain-containing 3 (SETD3) located on chromosome 14q32.2. It was designated circSETD3. To confirm the circular structure of circSETD3, three independent experiments were performed. We first inserted the PCR products of circSETD3 into the T vector for Sanger sequencing. As shown in Fig. 2a, the result of sequencing was consistent with the back-spliced region of circSETD3 supplied by circBASE [28]. In addition, we designed two sets of primers. One set comprised divergent primers for circular transcripts and the other set comprised convergent primers for linear transcripts. The two sets of primers were used to amplify the circular and linear transcripts of SETD3 in both cDNA and gDNA from HCC and paired non-tumorous tissues, as well as Hep3B cells. The circular transcripts were amplified by divergent primers in cDNA, but not in gDNA, while the linear transcripts could be amplified by convergent primers in both cDNA and gDNA. No product was amplified by divergent primers of GAPDH in cDNA and gDNA in the GAPDH negative control gene (Fig. 2b). The circular structure of circSETD3 was confirmed by RNase R experiment. As shown in Fig. 2c, the linear transcripts of SETD3 amplified from HCC tissues, paired non-tumorous tissues and HepG2 cells were obviously degraded by RNase R, while the circular transcripts of SETD3 were resistant to RNase R treatment. Taken together, the data demonstrated the circular structure of circSETD3.

### Correlation between circSETD3 expression and clinical characteristics of HCC

To further investigate the role of circSETD3 in HCC, the relationship between circSETD3 expression in HCC tissues and clinicopathological characteristics of HCC patients was analysed. As shown in Table 2, low expression of circSETD3 in HCC tissues was significantly correlated with larger tumour size (*P* = 0.01) and poorer tumour differentiation (*P* = 0.01), but not with other characteristics of HCC that included the level of alpha fetoprotein level, tumour number, Barcelona Clinic Liver Cancer (BCLC) stage, vascular invasion and other features. The data provide evidence of the importance of circSETD3 in the growth and tumorigenesis of HCC.

**Table 2:** The relationship between the expression of circ-SETD3 and the clinical characteristics of HCC patients

Parameters	Patient number (total = 132)	cric-SETD3 expression(2^-△△t^)	
		mean ± SD	*P* value
Gender			
Male	120	0.37 ± 0.20	0.81
Female	12	0.42 ± 0.42	
Age (years)			
≥60	64	0.33 ± 0.32	0.13
< 60	68	0.49 ± 0.46	
HBV-DNA			
≥10^3 copies/mL	71	0.36 ± 0.33	0.34
< 10^3 copies/mL	61	0.47 ± 0.48	
AFP			
≥400 ng/ml	64	0.40 ± 0.48	0.83
< 400 ng/ml	68	0.42 ± 0.33	
Child-Pugh Grade			
A	125	0.42 ± 0.41	0.46
B	7	0.24 ± 0.23	
Tumor number			
Multi	28	0.31 ± 0.43	0.33
Single	104	0.44 ± 0.40	
Tumor size			
≥5 cm	85	0.31 ± 0.32	0.01^*^
< 5 cm	47	0.59 ± 0.49	
Tumor capsule			
Complete	52	0.48 ± 0.35	0.32
Infiltrate	80	0.37 ± 0.44	
GVI			
Present	38	0.45 ± 0.55	0.64
Absent	94	0.40 ± 0.34	
BCLC stage			
A	28	0.58 ± 0.29	0.26
B	59	0.35 ± 0.33	
C	45	0.39 ± 0.53	
MVI			
Present	68	0.38 ± 0.46	0.57
Absent	64	0.44 ± 0.34	
Differetiation			
High + moderate	82	0.50 ± 0.46	0.01^*^
Moderate to low + low	50	0.26 ± 0.23	
Ishak score			
0–2	5	0.18 ± 0.07	0.21
3–4	54	0.32 ± 0.28	
5–6	73	0.49 ± 0.47	

*HCC* hepatocellular carcinoma, *AFP* alpha fetal protein, *HBsAg* hepatitis B surface antigen, *BCLC* Barcelona-Clinic Liver Cancer, *GVI* gross vascular invasion defined as the tumor embolus were observed in the first or second branches of the portal veins by preoperative radiological tests, such as CT or MRI, *MVI* microvascular invasion, circSETD3 circular RNA SETD3, *SD* standard deviation

*statistical significance

### CircSETD3 inhibits HCC growth in vitro

**Fig. 3:**
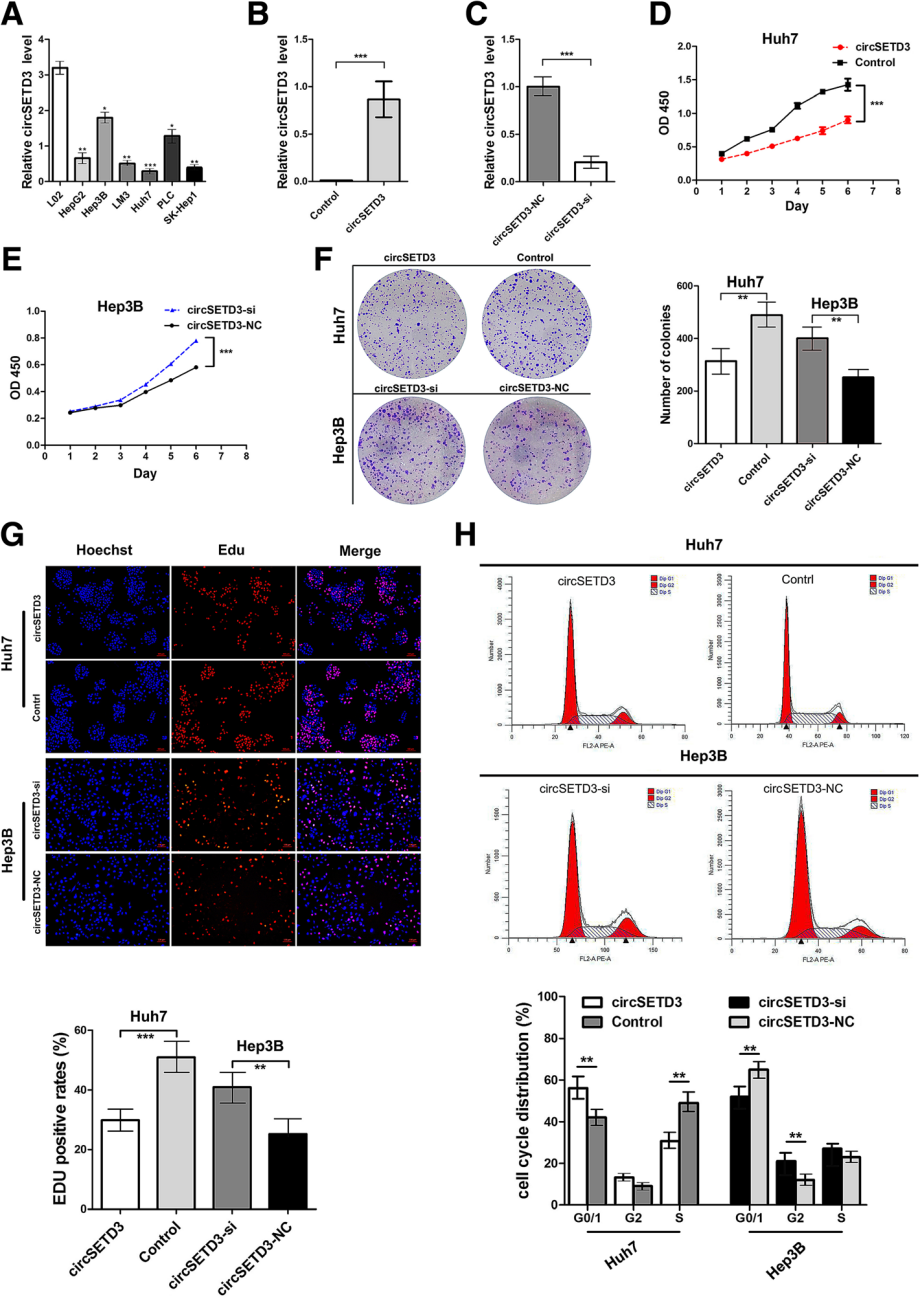
CircSETD3 inhibits the proliferation of HCC cells in vitro. **a** The expression levels of circSETD3 in multiple HCC cell lines. **b** The expression level of circSETD3 in Huh7 cells were successfully overexpressed by circSETD3 letivirus. **c** The expression level of circSETD3 in Hep3B cells were knocked down by circSETD3 siRNA. **d-g** CCK-8 (**d** and **e**), colony formation (**f**), and EdU (**g**) assays showed the overexpression of circSETD3 inhibited the growth of Huh7 cells while knockdown of circSETD3 promoted the growth of Hep3B cells. **h** Cell cycle was analysed using flow cytometry after transfection with circSETD3 letivirus or siRNA. Overexpression of circSETD3 induced G1/S arrest in Huh7 cells. Knockdown of circSETD3 relieved the G1/S arrest in Hep3B cells. HCC, hepatocellular carcinoma; NC, negative control. **P < 0.01, ***P < 0.001. Error bars indicate SD

The expression level of circSETD3 in multiple HCC and normal liver cell lines were measured. The expression level of circSETD3 in HCC cell lines was generally lower than that in the LO2 normal liver cell line (Fig. 3a). The expression of circSETD3 was lowest in Huh7 cells and highest in Hep3B cells. Using circSETD3-overexpressing lentivirus, the circSETD3 expression in Huh7 cells was shown to be dramatically increased (Fig. 3b). Using the backsplice junction-specific siRNA, we successfully knocked down circSETD3 expression in Hep3B cells (Fig. 3c). Analyses using CCK-8 (Fig. 3d and e), colony formation (Fig. 3f) and EdU proliferation assays (Fig. 3g) revealed that ectopic expression of circSETD3 significantly restrained the proliferation of Huh7 cells, while the knockdown of circSETD3 significantly promoted the proliferation of Hep3B cells. We further investigated whether circSETD3 affected cell cycle progression or apoptosis of HCC cells using flow cytometry. The ectopic expression of circSETD3 significantly increased the percent of Huh7 cells in G0/G1 phase and decreased the percent of Huh7 cells in S phase. The knockdown of circSETD3 significantly decreased the percent of Hep3B cells in G0/G1 phase and increased the percent of Hep3B cells in G2 phase (Fig. 3h). However, circSETD3 overexpression and knockdown did not affect apoptosis of HCC cells (Additional file 1: Figure S2). The collective findings suggested that circSETD3 inhibits the proliferation of HCC cells.

### CircSETD3 has no effect on its linear transcript

**Fig. 4:**
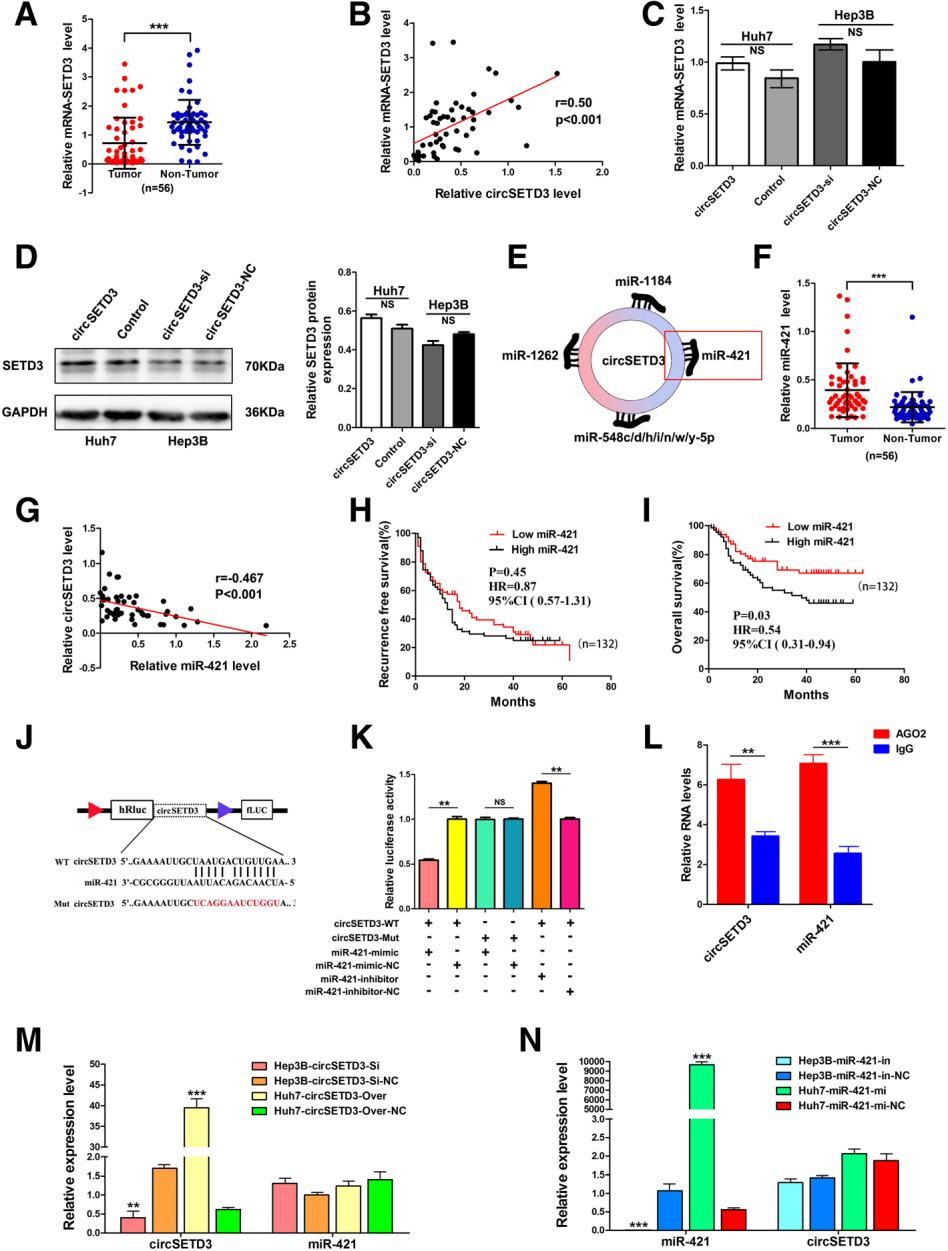
SETD3 is not the target of circSETD3 whereas circSETD3 acts as a sponge for miR-421 in HCC. **a** The expression level of SETD3 mRNA in 56 pairs of HCC and non-tumorous tissues. **b** CircSETD3 positively correlated with SETD3 mRNA in HCC tissues. **c** and **d** The mRNA and protein levels of SETD3 were not changed after artificially changed the expression of circSETD3 in HCC cells. **e** 10 most possible miRNAs that could be sequestered by circSETD3 were identified. Among them, miR-421 is a well-demonstrated tumor promoter. **f** miR-421 was significantly upregulated in HCC tissues compared with matched non-tumorous tissues. **g** The expression of miR-421 was negatively associated with circSETD3 in HCC tissues. **h** and **i** Kaplan-Meier’s survival curves depicted the correlations between miR-421 and RFS or OS of HCC patients (the patients were stratified into two groups according to median of Mr-421). Log-rank test was used. **j** Schematic of circSETD3 wild-type (WT) and mutant (Mut) luciferase reporter vectors. **k** The relative luciferase activities were analyzed in 293 T cells co-transfected with miR-421 mimics, miR-mimics-NC, miR-421 inhibitors or miR-inhibitors-NC and WT or Mut luciferase reporter vectors. **l** The Ago2 RIP showed that Ago2 significantly enriched circSETD3 and miR-421. **m** Expression change of circSETD3 did not affect the expression of miR-421. **n** Expression change of miR-421 did not affect the expression of circSETD3. HCC, hepatocellular carcinoma; NS, not significant; RIP, RNA immunoprecipitation. **P < 0.01, ***P < 0.001. Error bars indicate SD

Some circRNAs regulate the expression and function of the corresponding linear transcripts [20, 29, 30]. Therefore, the regulatory relationship between circSETD3 and its linear transcript (SETD3) was investigated in this study. We first examined the expression level of SETD3 in 56 paired HCC and adjacent non-tumorous tissues. SETD3 was significantly downregulated in HCC tissues compared with adjacent non-tumorous tissues (Fig. 4a). Pearson’s correlation analysis revealed a significant positive correlation between circSETD3 and SETD3 in HCC tissues (Fig. 4b). However, SETD3 did not change in both mRNA and protein levels when the expression of circSETD3 was artificially changed in HCC cells (Fig. 4c and d). These data indicated that SETD3 is not the target gene of circSETD3.

### CircSETD3 acts as a sponge for miR-421

Given that exonic circRNAs are stable and are normally localized in the cytoplasm, in which circRNAs function as an miRNA sponge to regulate gene expression [31], we next investigated the miRNAs that could potentially interact with circSETD3. The miRanda database and mirSVR algorithm were used to identify the 10 miRNAs that could most likely be sequestered by circSETD3 (Fig. 4e and Additional file 1: Figure S3). Among the 10 miRNAs, seven were family members of miR-548. The remaining three were miR-421, miR-1184 and miR-1262. Prior studies had shown that only miR-421 is upregulated in HCC tissues and functions as a tumour promoter in HCC [32], and that the high expression of miR-421 in HCC tissues predicts a poor prognosis of HCC patients [33]. Therefore, in the present study, the clinical significance of miR-421 in HCC patients and the regulatory relationship between miR-421 and circSETD3 were analysed.

Consistent with the previous studies, the expression levels of miR-421 in the 56 HCC samples were significantly higher than that in paired non-tumorous samples (Fig. 4f). Furthermore, miR-421 was negatively correlated with circSETD3 in HCC tissues (Fig. 4g). Subsequently, a survival analysis was performed using a larger cohort that included 132 HCC patients allocated into high and low groups based on the median value of miR-421 expression. High expression of miR-421 was significantly associated with poor OS (Fig. 4i) other than RFS (Fig. 4h) of HCC patients. These results demonstrated that miR-421 is indeed an important tumour promoter of HCC. Next, to verify the regulatory relationship between circSETD3 and miR-421, we constructed two luciferase reporters containing wild-type and mutant circSETD3 (the predicted miR-421 binding site was mutated) (Fig. 4j). Compared with control RNA, the luciferase activity was significantly reduced when cells were co-transfected with miR-421 mimics with the wild-type luciferase reporter. However, the luciferase activity was significantly increased when cells were co-transfected with miR-421 inhibitors with the wild-type luciferase reporter. The luciferase activity did not significantly change when cells were co-transfected miR-421 mimics or inhibitors with mutant luciferase reporter (Fig. 4k). A previous study reported that Argonaute 2 (Ago2) protein binds with both circRNAs and miRNAs to form the RNA-induced silencing complex. Therefore, an RNA immunoprecipitation (RIP) assay was presently performed to pull down RNA transcripts bound to Ago2 in Huh7 cells. As expected, both circSETD3 and miR-421 were efficiently pulled down by anti-Ago2, but not by the non-specific anti-IgG antibody (Fig. 4l). Additionally, overexpressing or silencing circSETD3 did not affect the expression of miR-421 (Fig. 4m), and the expression of circSED3 also did not change after transfection with miR-421 mimics or inhibitors (Fig. 4n). These findings indicated that circSETD3 acts as a sponge for miR-421.

### CircSETD3 inhibits HCC growth through the circSETD3/miR-421/MAPK14 pathway

**Fig. 5:**
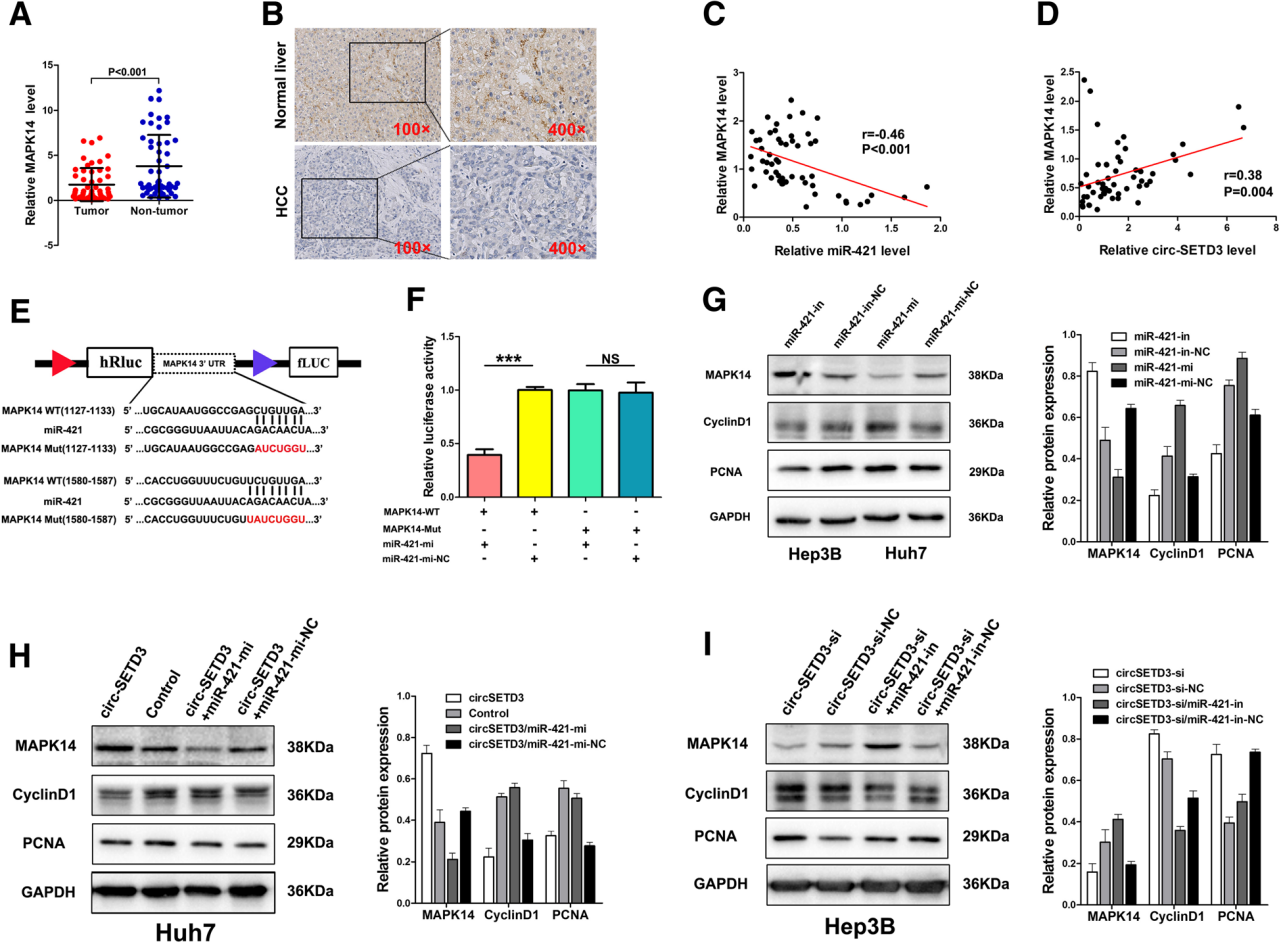
CircSETD3 inhibits the growth of HCC through thecircSETD3/miR-421/MAPK14 pathway. **a** and **b** Both qRT-PCR and IHC showed MAPK14 was significantly downregulated in HCC tissues compared with matched non-tumorous tissues. **c** and **d** MAPK14 negatively correlated with miR-421 whereas positively correlated with circSETD3 in HCC tissues. **e** Schematic of MAPK14 wild-type (WT) and mutant (Mut) luciferase reporter vectors. **f** The relative luciferase activities were analyzed in 293 T cells co-transfected with miR-421 mimics or miR-mimics-NC and WT or Mut luciferase reporter vectors. **g** MiR-421 inhibitor up-regulated MAPK14 and down-regulated cyclinD1 and PCNA in Hep3B cells. MiR-421 mimics down-regulated MAPK14 and up-regulated cyclinD1 and PCNA in Huh7 cells. **h** CircSETD3 letivirus up-regulated MAPK14 and down-regulated cyclinD1 and PCNA in Huh7 cells, this effect could be reversed by co-transfected with miR-421 mimics. **i** circSETD3 siRNA down-regulated MAPK14 and up-regulated cyclinD1 and PCNA in Hep3B cells, this effect can be reversed by co-transfected with miR-421 inhibitors. HCC, hepatocellular carcinoma; qRT-PCR, quantitative reverse transcription polymerase chain reaction; in, inhibitors; mi, mimics; IHC, immunohistochemistry. ***P < 0.001. Error bars indicate SD

Given that miRNAs could play an important role in post-transcriptional gene regulation by partially base-paring with the 3′-untranslated region (3′UTR) of target mRNAs, the target mRNAs of miR-421 were predicted using four bioinformatic logarithms: miRWalk [34], miRanda [35], RNA22 [36], and Targetscan [37]. MAPK14, a well-known tumour suppressor of HCC, displayed two binding sites for miR-421 (Fig. 5e). Using qRT-PCR and IHC, we found that the mRNA and protein expression levels of MAPK14 in HCC tumour tissues were significantly lower than those in the paired non-tumorous tissues (Fig. 5a and b). Pearson’s correlation analysis revealed that MAPK14 was negatively associated with miR-421 and positively associated with circSETD3 (Fig. 5c and d). To further confirm the relationship between miR-421 and MAPK14, a dual-luciferase reporter assay was performed. Luciferase activity was significantly reduced when 293 T cells were co-transfected with miR-421 mimics and a luciferase reporter containing wild-type 3′ UTR of MAPK14. However, the luciferase activity did not change significantly when 293 T cells were co-transfected with miR-421 mimics and a luciferase reporter containing a MAPK14 3′ UTR in which the predicted miR-421 binding site was mutated (Fig. 5f). We found that miR-421 inhibitors increased the protein levels of MAPK14, whereas miR-421 mimics decreased the protein levels of MAPK14 in HCC cells (Fig. 5g). The protein levels of Cyclin D1 and proliferating cell nuclear antigen (PCNA), which were used as indicators of cellular proliferation, were also altered accordingly. Furthermore, to confirm whether circSETD3 inhibits cell proliferation by targeting the miR-421/MAPK14 pathway, we demonstrated that the overexpression of circSETD3 upregulated MAPK14 protein level, whereas the silencing of circSETD3 downregulated the level of MAPK14 protein. The protein levels of Cyclin D1 and PCNA were also changed accordingly. The effects of circSETD3 on MAPK14, Cyclin D1, and PCNA could be reversed by miR-421 in HCC cells (Fig. 5h and i). Taken together, the data indicate that circSETD3 mediated inhibition of HCC growth might be partly linked to the circSETD3/miR-421/MAPK14 pathway.

### Stably existing circSETD3 inhibits HCC growth in vivo by targeting MAPK14

**Fig. 6:**
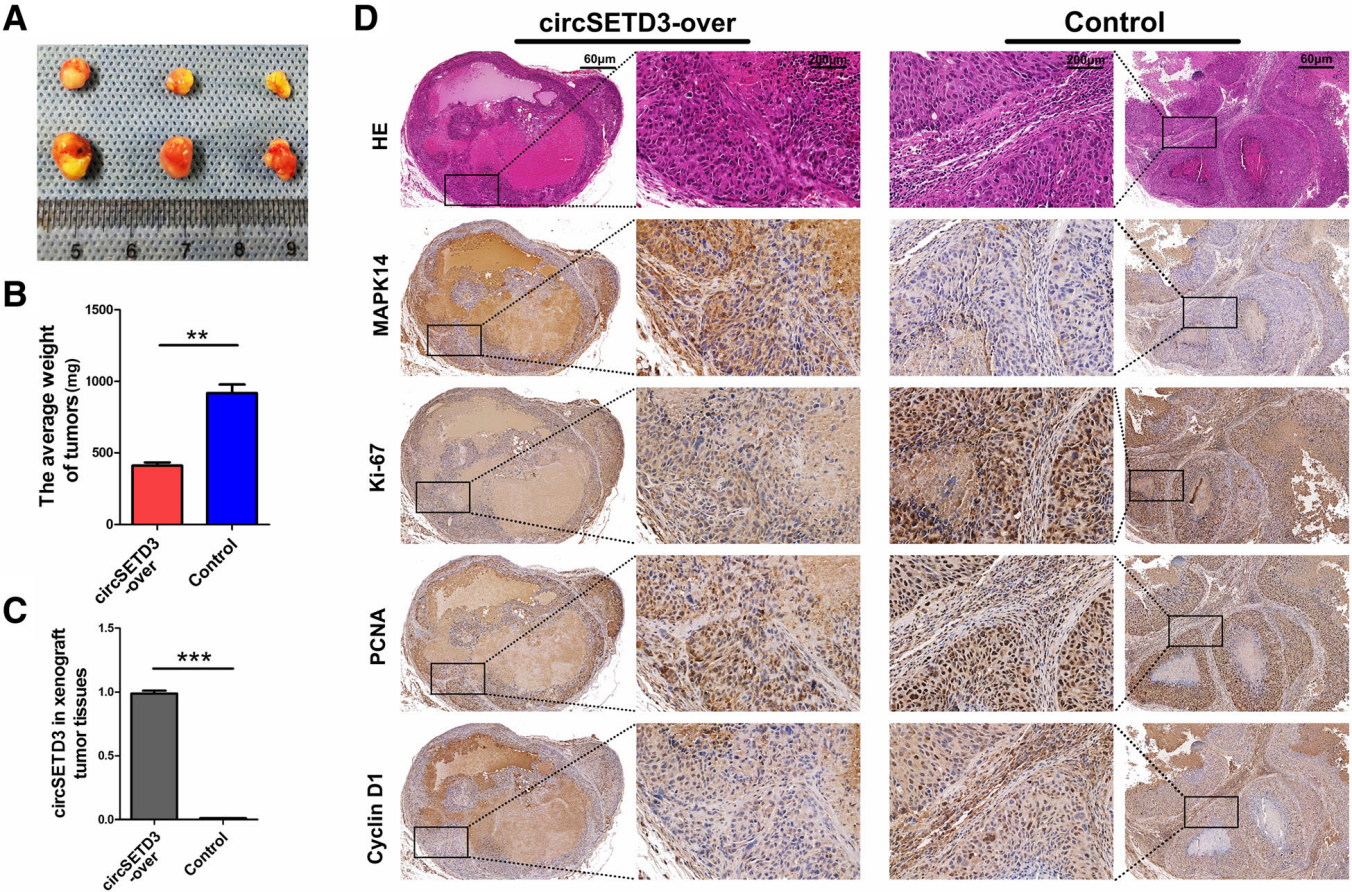
CircSETD3 stably maintained in xenograft tumor models and inhibit tumor growth by targeting MAPK14. **a** and **b** Smaller tumor size and lower tumor weight were observed in circSETD3-overexpressing group. **c** Over-expressed circSETD3 could stably maintained in xenograft tumor models. **d** The expression of MAPK14 was increased, Ki-67, PCNA and Cyclin D1 were decreased in circSETD3-overexpressing group when compared with control group. **P < 0.01, ***P < 0.001. Error bars indicate SD

To verify the effect of circSETD3 on the regulation of HCC growth in vivo, Huh7 cells stably transfected with circSETD3-overexpressing lentivirus or control lentivirus were subcutaneously injected into nude mice. Compared with the control group, smaller tumour size and lower tumour weight were observed in the circSETD3 overexpression group (Fig. 6a, b and c). RT-PCR revealed that the overexpressed circSETD3 could be stably maintained in the xenograft tumour model (Fig. 6d). IHC staining results showed that overexpressed circSETD3 significantly promoted the expression of MAPK14, while inhibiting the expression levels of Ki-67, PCNA and Cyclin D1 when compared with the control group (Fig. 6e). These results demonstrated that circSETD3 can inhibit the growth of HCC in vivo partly by regulating MAPK14.

## Discussion

Since the demonstration of the stable expression of numerous circRNAs in eukaryocytes and the fact that some of them possess strong miRNA-binding capability [13, 14], interest in the role of circRNAs in a variety of diseases has risen. Recently, Wang et al. [38] reported that circSETD3 was downregulated in colorectal cancer and served as a potential diagnostic biomarker. However, the expression profile of circSETD3 in HCC and its underlying mechanism remain unknown. In this study, data obtained through microarray and qRT-PCR analyses were combined with the information of HCC recurrence to identify the significant downregulation of circSETD3 in HCC tissues and its association with HCC prognosis. The downregulation of circSETD3 in HCC tissues was significantly associated with larger tumour size and unfavourable differentiation of HCC, indicating that circSETD3 functions as a tumour suppressor and might be associated with the growth and carcinogenesis of HCC. This presumption was subsequently validated in vitro and in vivo. Moreover, we demonstrated that circSETD3 acts as a sponge for miR-421 and identified MAPK14 as a novel target of miR-421. Taken together, our data demonstrate that circSETD3 inhibits the growth and tumorigenesis of HCC partly via the circSETD3/miR-421/MAPK14 axis.

SETD3 is transcribed from Chr14q32.2, which is a critical region frequently involved in tumorigenesis [39]. As a conserved histone H3 methyltransferase, SETD3 is abundantly expressed in many tissues and plays different roles. In muscle, it promotes myocyte differentiation by regulating the expression of myogenin through interaction with MyoD [40]. In renal cell tumours, SETD3 is mainly observed in the cytoplasm and the downregulation of SETD3 is significantly associated with shorter disease-specific and disease-free survival [41]. In lymphoma, a truncated variant of SETD3 lacking the SET domain sequences is highly expressed in the lymphoma and promotes lymphomagenesis [42]. In HCC, SETD3 is predominantly localized in the cytoplasm and only a small portion of SETD3 is sporadically found in the nucleus [43]. The protein levels of SETD3 are significantly higher in HCC tissues than in adjacent normal tissues, and SETD3 levels are positively correlated with the proliferation of HCC. However, the mRNA levels of SETD3 in tumours are lower than those in normal tissues based on The Cancer Genome Atlas (TCGA). In the present study, the mRNA expression level of SETD3 in 56 pairs of HCC and matched non-tumorous tissues were measured using qRT-PCR. Consistent with TCGA data, SETD3 mRNA was significantly downregulated in HCC tissues compared with the matching non-tumorous tissues, and mRNA-SETD3 expression levels in HCC tissues were positively correlated with circSETD3. These observations indicate that the expression of circSETD3 in HCC may be regulated mainly by the amplification of host gene. Recently, Bai et al. [30] and He et al. [29] reported that circFBLIM1 and circGRFA1 can regulate corresponding linear transcripts expression via a ceRNA mechanism. However, we found that both the mRNA and protein levels of SETD3 did not significantly change after artificially regulating the circSETD3 expression in HCC cells, indicating a lack of regulatory relationship between circSETD3 and its linear transcript.

Previous studies have reported that circRNAs arising from exonic regions typically reside in the cytoplasm and some may serve as miRNA sponges [14]. In the present study, we also demonstrated that circSETD3 could bind to miR-421, a well-studied tumour promoter in multiple cancers. Hu et al. [44] found that miR-421 was upregulated in breast cancer tissues and inhibited cell apoptosis by restraining capase-10 expression. Yang et al. [45] demonstrated that miR-421 promote the proliferation, invasion and metastasis of gastric cancer by inhibiting the expression of CLDN11. Chen et al. [46] reported that miR-421 played an oncogenic role in nasopharyngeal carcinoma. In HCC, Zhang et al. [32] described that miR-421 could regulate the expression of farnesoid X receptor to promote the proliferation, migration and invasion of HCC cells. Subsequently, using TCGA information, Lu et al. [33] identified that miR-421 was significantly upregulated in HCC samples compared with non-cancer samples, and high expression of miR-421 in HCC tissues predicted an unfavourable OS in patients with HCC. These results are very consistent with the present findings. Taken together, the collective evidence indicates that miR-421 has essential roles in various cancers, so it is not difficult to presume that circSETD3 may be also a key factor of tumours because it is a sponge for miR-421.

Additionally, we also predicted and verified that MAPK14 is a novel target of miR-421. MAPK14, which is also termed p38 MAPK, is a serine/threonine kinase that is mainly associated with stress responses and apoptosis [47, 48]. However, MAPK14 may also act on cell cycle checkpoints and can function as a suppressor of cell proliferation and tumorigeneses [49, 50]. In this study, the expression of MAPK14 could be regulated by miR-421, and these effects could be efficiently reversed by circSETD3. These observations indicate that circSETD3 could protect MAPK14 from miR-421-mediated degradation via a ceRNA mechanism.

## Conclusions

The data presented herein implicate circSETD3 as a candidate tumour suppressor of HCC. Also, circSETD3 could be a useful prognostic biomarker in patients with HCC who have received curative liver resection. Moreover, circSETD3 inhibits the growth and tumorigenesis of HCC partly by targeting the miR-421/MAPK14 pathway. Taken together, these data indicate that circSETD3 might be a novel biomarker and potential therapeutic target for HCC.

## Additional file

Additional file 1: **Figure S1.** The relationship between the expression level of selected circRNAs and recurrence-free survival of HCC patients. **Figure S2.** Cell apoptosis analysis after transfection with circSETD3 letivirus or siRNA. **Figure S3.** The predicted circSETD3 targeted circRNA-miRNA-mRNAs network based on sequence-paring prediction. (DOCX 977 kb)

## Abbreviations

circRNAs: Circular RNAs
circSETD3: Circular RNA SETD3
HCC: Hepatocellular carcinoma
MVI: Microvascular invasion
OS: Overall survival
qRT-PCR: Quantitative real-time PCR
RFS: Recurrence-free survival
RIP: RNA immunoprecipitation
SETD3: SET domain-containing 3
