# Supplementary material for: CircSETD3 (Hsa_circ_0000567) acts as a sponge for microRNA-421 inhibiting hepatocellular carcinoma growth
Source: J Exp Clin Cancer Res. 2019 Feb 22;38:98. doi: 10.1186/s13046-019-1041-2 (PMC6385474; doi:10.1186/s13046-019-1041-2)
Supplement: Supplementary file 1 — Figure S1. The relationship between the expression level of selected circRNAs and recurrence-free survival of HCC patients. Figure S2. Cell apoptosis analysis after transfection with circSETD3 letivirus or siRNA. Figure S3. The predicted circSETD3 targeted circRNA-miRNA-mRNAs network based on sequence-paring prediction. (DOCX 977 kb) [file 13046_2019_1041_MOESM1_ESM.docx]

**Figure S1**

**
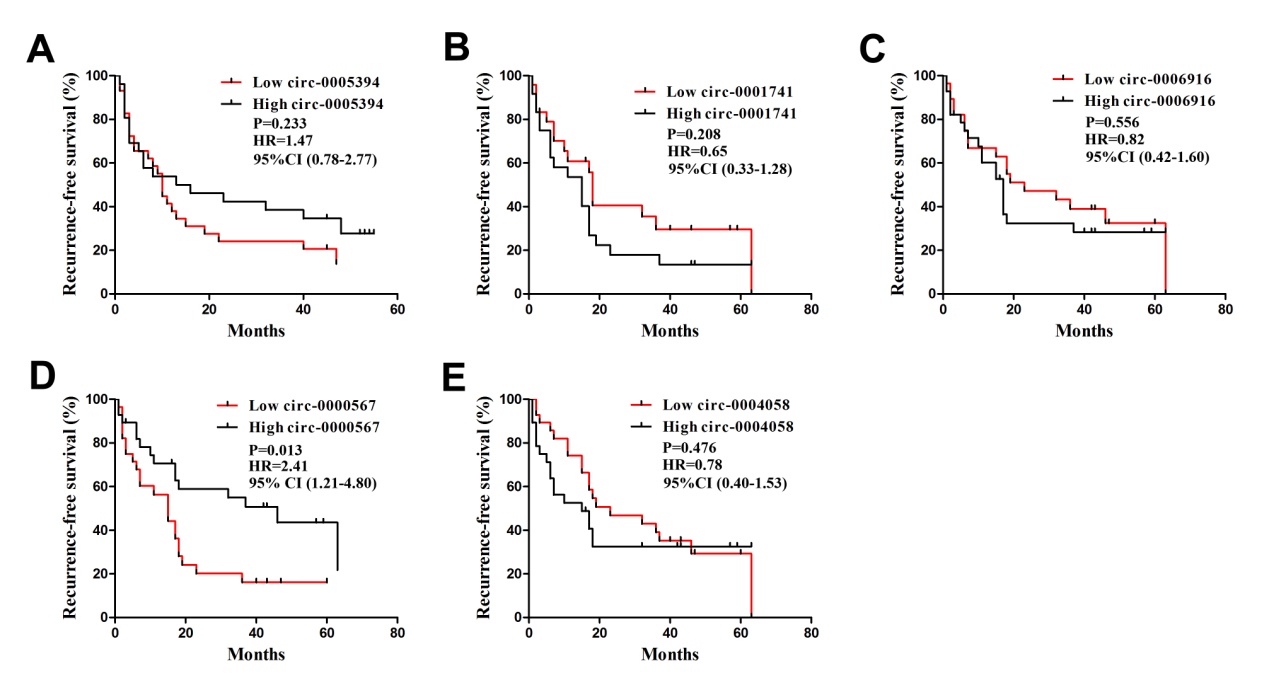
**

**Fig. S1 The relationship between the expression level of selected circRNAs and recurrence-free survival of HCC patients. a.** The patients were divided according to the expression level of hsa_circ_0005394. **b.** The patients were divided according to the expression level of hsa_circ_0001741. **c.** The patients were divided according to the expression level of hsa_circ_0006916. **d.** The patients were divided according to the expression level of hsa_circ_0000567 (circSETD3). **e.** The patients were divided according to the expression level of hsa_circ_0004058.

**Figure S2**

**
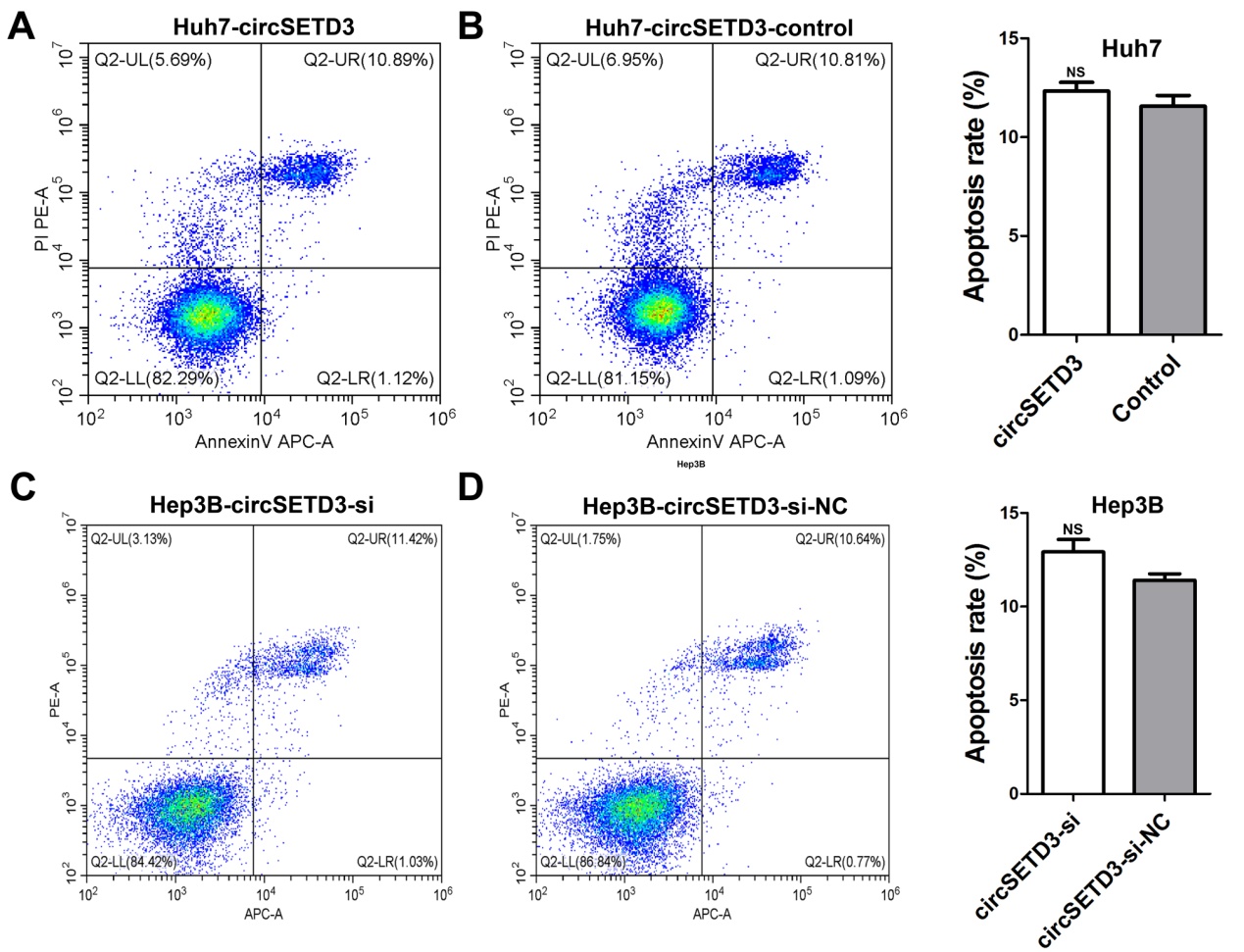
**

**Fig. S2 Cell apoptosis analysis after transfection with circSETD3 letivirus or siRNA. a** and **b**. Huh7 cells transfected with circSETD3 letivirus or control letivirus. **c** and **d**. Hep3B cells transfected with circSETD3 siRNA or negative control siRNA. NC, negative control.

**Figure S3**

**
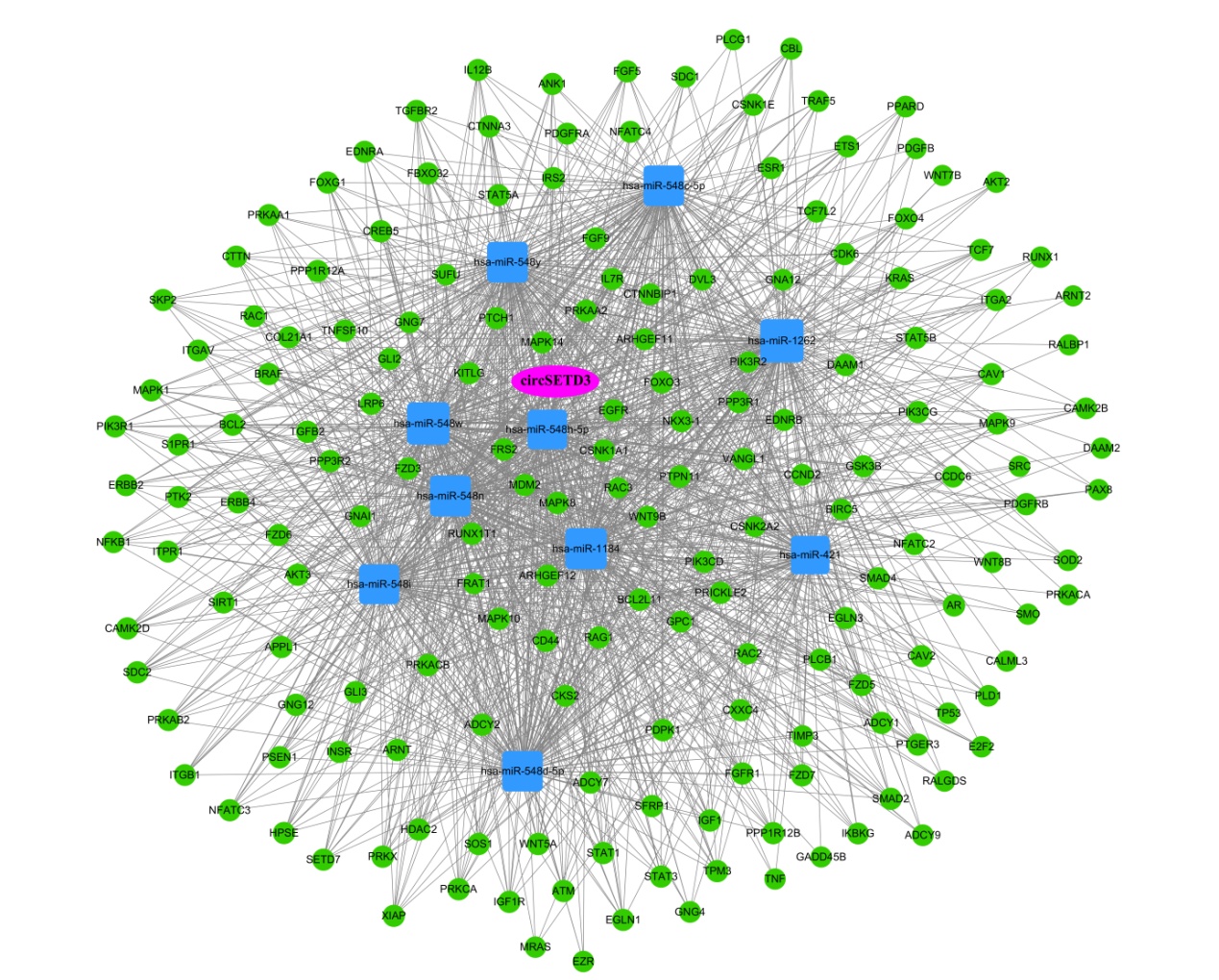
**

**Fig. S3 The predicted circSETD3 targeted circRNA-miRNA-mRNAs network based on sequence-paring prediction.** The potential miRNA-binding sites were predicted by mirSVR, and targeted miRNAs and mRNAs were predicted by four bioinformatic logarithms. As a results, 10 miRNAs and related mRNAs were included in this network.
